# Endovascular Embolization for Control of Post-Tonsillectomy Hemorrhage

**DOI:** 10.7759/cureus.13217

**Published:** 2021-02-08

**Authors:** Alanna M Windsor, Liuba Soldatova, Lisa Elden

**Affiliations:** 1 Otorhinolaryngology - Head and Neck Surgery, Montefiore Medical Center, Bronx, USA; 2 Otorhinolaryngology - Head and Neck Surgery, University of Pennsylvania Perelman School of Medicine, Philadelphia, USA; 3 Otolaryngology, Children’s Hospital of Philadelphia, Philadelphia, USA

**Keywords:** tonsillectomy, interventional radiology, endovascular procedures, post-tonsillectomy hemorrhage

## Abstract

Post-operative hemorrhage is a potentially life-threatening complication of tonsillectomy. While standard surgical maneuvers including the use of electrocautery, application of topical hemostatic agents, direct pressure, and suturing of the tonsillar pillars have traditionally been used for the treatment of severe bleeding, endovascular approaches are an important adjunct when other techniques are unsuccessful. Here, we describe the case of a 10-year-old female who presented with severe bleeding four days after tonsillectomy and adenoidectomy for chronic tonsillitis. She was taken emergently to the operating room where pulsatile bleeding was noted from the right inferior tonsillar pole. Hemostasis could not be achieved using electrocautery despite multiple attempts. The patient was taken for emergent angiography, which demonstrated an irregularity of the right tonsillar artery consistent with arterial vasospasm, and which corresponded to the intraoral site of bleeding localized by the surgeon. Coil embolization of the tonsillar artery was successfully performed, and the patient experienced no further bleeding. We conclude that endovascular embolization of branches of the external carotid artery is an effective treatment for severe post-tonsillectomy hemorrhage in children and should be considered when attempts at surgical control are ineffective. This procedure requires exceptional collaboration between the surgical, radiology, and anesthesia teams.

## Introduction

Tonsillectomy is one of the most common surgical procedures performed in the United States [1]. Post-operative hemorrhage is a serious risk as the palatine tonsil is a highly vascularized region that receives blood supply from branches of the lingual, facial, and maxillary arteries. Rates of post-tonsillectomy bleeding of up to 3% have been reported in the literature [1]. Here, we describe a case of post-tonsillectomy bleeding in a pediatric patient who was refractory to standard surgical maneuvers and was effectively managed through endovascular embolization of the tonsillar branch of the facial artery.

This work was previously presented as a poster at the American Society of Pediatric Otolaryngology Summer Meeting, July 2019, Vail, CO.

## Case presentation

A 10-year-old female with a history of obesity presented to the emergency room four days after tonsillectomy and adenoidectomy at another hospital for chronic tonsillitis. Her parents reported multiple episodes of coughing up large volumes of blood at home. On arrival, she was hemodynamically stable and her hemoglobin was 11.1 g/dL. Physical examination demonstrated a large clot in the right tonsillar fossa. Given the patient’s physical examination findings and history consistent with repeated severe bleeding episodes, the patient was taken urgently to the operating room (OR) to identify and control the source of bleeding. Her airway was secured with an endotracheal tube and the tonsillar fossa was exposed using a mouth gag. Pulsatile bleeding was encountered in the right inferior tonsillar pole. Electrocautery was used at the site of bleeding; however, the vascular source could not be controlled with multiple attempts as it retracted into the surrounding inflamed tissues. The surgical team, therefore, opted to pack the patient’s oropharynx and transfer her to the interventional radiology suite for diagnostic angiography with possible embolization.

**Figure 1:**
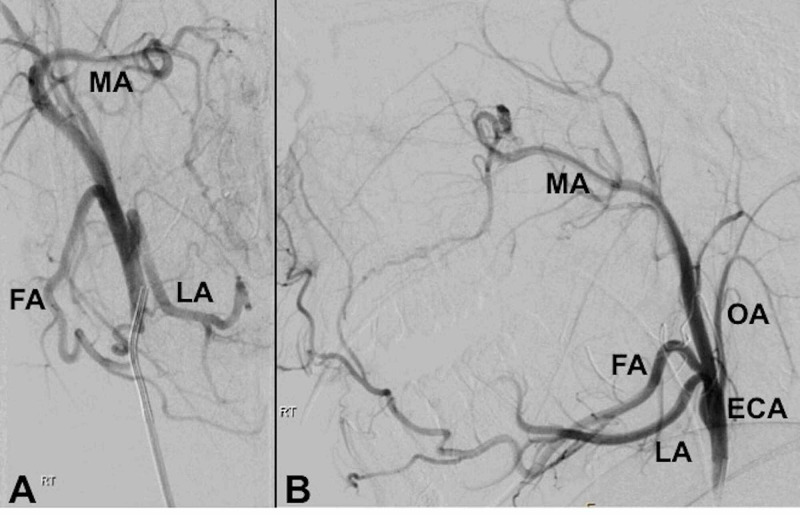
Pre-embolization angiogram of the right external carotid in the antero-posterior (A) and lateral (B) projections. MA, maxillary artery; FA, facial artery; LA, lingual artery; ECA, external carotid artery; OA, occipital artery

**Figure 2:**
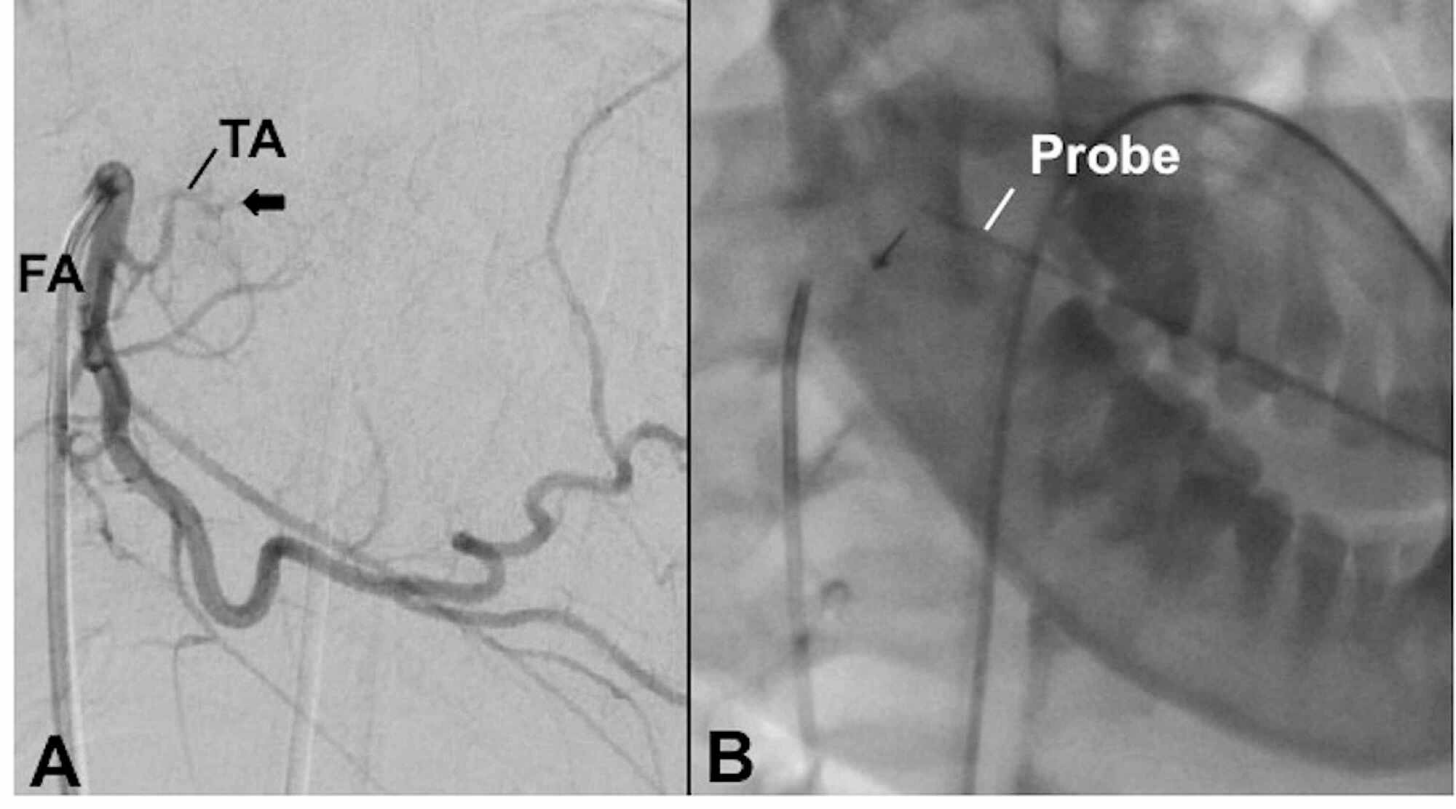
Selective angiography of the right FA in the lateral projection. (A) Irregularity and abnormal truncation of the TA artery is seen, as indicated by the arrow. No active extravasation is noted. (B) This site corresponded to the location of a radiopaque probe used by the surgeon to indicate the site of bleeding transorally during angiogram. FA, facial artery; TA, tonsillar artery

A 5 Fr catheter was introduced via the right common femoral route and used to select the right external carotid artery. Angiographic assessment was made of the right external carotid artery (Figures 1A, 1B). The catheter was then advanced into the right facial artery (Figure 2A). The tonsillar branch was noted to have an irregularity and abnormal truncation of its course, consistent with transient vasospasm. No active extravasation was noted. Under direct visualization, the otolaryngologist used a radiopaque probe to manually identify the source of bleeding through the mouth at the inferior tonsillar pole (Figure 2B). The site of the bleeding indicated by the surgeon appeared to correspond to the location of the abnormal tonsillar artery.

 

**Figure 3:**
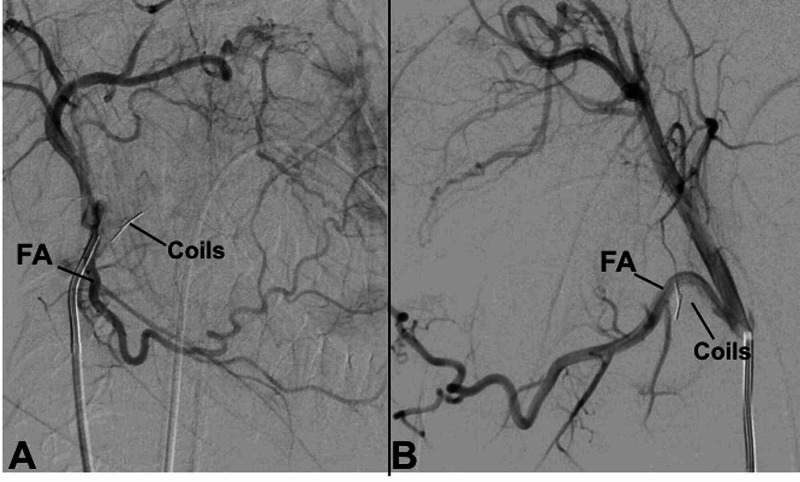
Post-embolization angiography of the right FA in the antero-posterior (A) and lateral (B) projections, demonstrating the coils in satisfactory position. FA, facial artery

A 2.5 Fr Renegade microcatheter (Boston Scientific, Marlborough, MA, USA) was used to sub-select the tonsillar artery. Two 0.018" 0.5 cm straight Hilal coils (COOK Medical, Bloomington, IN, USA) were deployed into the right tonsillar artery (Figures 3A, 3B). The surgeon then performed manual aggressive agitation of the bleeding site. Subsequent angiography of the right facial artery demonstrated no active extravasation, and the patient had no active intraoral bleeding on examination. After the procedure, the patient was transferred to the intensive care unit. She was monitored and had no further bleeding for the duration of her hospitalization. She was discharged home in stable condition on post-operative day five.

## Discussion

While tonsillectomy is generally a safe procedure, bleeding is a well-recognized complication that can lead to significant morbidity and even mortality. Patients who undergo tonsillectomy for an indication of chronic tonsillitis, as in our patient, may have an increased risk of post-operative hemorrhage and may be more likely to require surgical intervention in the OR [2]. Post-tonsillectomy bleeding may be managed with observation, bedside interventions, or surgery. Maneuvers to control bleeding include electrocautery, application of topical hemostatic agents, direct pressure to the site of bleeding, and suturing of the tonsillar pillars [3,4]. Substantial variation exists in the management of post-tonsillectomy bleeding, though at our institution, surgical intervention is generally performed in cases of active bleeding, hemodynamic instability, significant drop in hemoglobin, multiple episodes of bleeding, or history of a large-volume bleed with evidence of clot on oropharyngeal examination. Furthermore, factors such as patient age, bleeding severity and duration, laboratory studies, and distance that the patient resides from the hospital may play a role in management [3]. In cases of severe bleeding that are refractory to standard surgical interventions, open ligation of branches of the external carotid artery and endovascular intervention may be considered.

Previous reports have described endovascular embolization of branches of the external carotid artery as an effective treatment for severe post-tonsillectomy hemorrhage when attempts at surgical control have failed [5,6]. In particular, endovascular treatment may be useful in cases of delayed post-tonsillectomy bleeding by identifying rare causes of bleeding such as vascular tears or arterial pseudoaneurysm [7-9]. Advantages of endovascular treatment over external carotid ligation include (1) the ability to perform a diagnostic angiogram and perform intervention during the same procedure, (2) the possibility of identifying and individually targeting feeding arteries to the tonsillar fossa, and (3) the avoidance of a neck incision [5,8]. Nonetheless, endovascular intervention is not without risks, and therefore, should be reserved for the most severe cases of bleeding in which transoral interventions have failed. Potential risks of endovascular interventions include vessel perforation leading to extravasation of embolic material or contrast, ischemic injury to mucosal surfaces and cranial nerves, inadvertent embolization of the internal carotid artery, or arterial vasospasm [5].

Our patient highlights several unique considerations in the use of this procedure to treat post-tonsillectomy hemorrhage. First, active participation by the surgeon during the endovascular procedure was crucial in identifying and confirming the site of bleeding, particularly because no active extravasation was visualized on angiography. In our patient, the surgeon was able to assist the radiologist in localizing the site of bleeding transorally using a radiopaque probe. This site corresponded to a subtle abnormality on angiography, but this maneuver increased the team’s confidence that the true source of bleeding had been identified. The surgeon also performed manual irritation of the site of bleeding after coil deployment to assess any additional sources of bleeding and to ensure appropriate hemostasis had been achieved. Second, this approach required the ability to rapidly activate the on-call interventional radiology team, including all supporting staff. At our institution, this team was mobilized within approximately one hour while the patient’s bleeding was temporized through the use of oropharyngeal packing. Finally, this procedure required excellent communication and collaboration between the surgeon, interventional radiologist, and the anesthesiologist to ensure the safety of the patient’s airway and hemodynamics.

## Conclusions

Endovascular embolization of the branches of the external carotid artery is an effective treatment for severe post-tonsillectomy hemorrhage. This procedure should be considered when standard surgical maneuvers, such as electrocautery, application of topical hemostatic agents, and direct pressure, are ineffective. This procedure requires exceptional collaboration between the surgical, radiology, and anesthesia teams to ensure the patient’s safety, successful identification of the bleeding source, and bleeding control.
